# Prevalence and factors associated with carriage of *Pfmdr1* polymorphisms among pregnant women receiving intermittent preventive treatment with sulfadoxine-pyrimethamine (IPTp-SP) and artemether-lumefantrine for malaria treatment in Burkina Faso

**DOI:** 10.1186/s12936-020-03473-5

**Published:** 2020-11-10

**Authors:** Hamtandi Magloire Natama, Rouamba Toussaint, Djamina Line Cerine Bazié, Sékou Samadoulougou, Maminata Coulibaly-Traoré, Halidou Tinto, Fati Kirakoya-Samadoulougou

**Affiliations:** 1 Unité de Recherche Clinique de Nanoro, Institut de Recherche en Sciences de la Santé, Nanoro, Burkina Faso; 2 Centre D’Epidémiologie, Biostatistique Et Recherche Clinique, Ecole de Santé Publique, Université Libre de Bruxelles (ULB), Bruxelles, Belgium; 3 Centre for Research On Planning and Development (CRAD), Laval University, Quebec, Canada; 4 Evaluation Platform On Obesity Prevention, Quebec Heart and Lung Institute, Quebec, Canada

**Keywords:** Malaria, Pregnancy, Artemether-lumefantrine, *pfmdr1* mutations

## Abstract

**Background:**

Single nucleotide polymorphisms occurring in the *Plasmodium falciparum* multidrug resistant gene 1 (*pfmdr1)* are known to be associated with aminoquinoline resistance and, therefore, represent key *P. falciparum* markers for monitoring resistance both in susceptible groups (children under 5 years old and pregnant women) and in the general population. This study aimed to determine prevalence and factors associated with the carriage of *pfmdr1* N86Y, Y184F and D1246Y polymorphisms among pregnant women in a setting of high malaria transmission in Burkina Faso.

**Methods:**

*Plasmodium falciparum* isolates were collected at the first antenatal care visit (ANC-1) as well as at delivery from pregnant women participating in the COSMIC trial (NTC01941264), which assessed malaria preventive interventions during pregnancy in the Nanoro Health District. Here, pregnant women received intermittent preventive treatment with sulfadoxine-pyrimethamine (IPTp-SP) and malaria infections and/or diseases were treated using artemether-lumefantrine (AL) during the trial. Parasite DNA was extracted from dried blood spots and the presence of *pfmdr1* mutations at positions 86, 184 and 1246 was determined using nested PCR, followed by restriction fragment length polymorphism (RFLP) analysis.

**Results:**

A prevalence of 13.2% (20/151) and 12.1% (14/116) of the *pfmdr1* 86Y mutant allele was found at ANC-1 and at delivery, respectively, while no mutant allele was observed for Y184F and D1246Y codons at both ANC-1 and at delivery. There were no significant factors associated with *pfmdr1* 86Y mutant allele carriage at ANC-1. However, malaria infections at delivery with a parasite density above the median (2237.2 (IQR: 613.5–11,425.7) parasites/µl) was associated with an increase risk of *pfmdr1* 86Y mutant allele carriage (AOR = 5.5 (95% CI  1.07–28.0); *P* = 0.04). In contrast, both three or more IPTp-SP doses (AOR = 0.25 (95% CI 0.07–0.92); *P* = 0.04) and one or more AL treatment (AOR = 0.25 (95% CI 0.07–0.89); *P* = 0.03) during pregnancy were associated with a significant reduce risk of *pfmdr1* 86Y mutant allele carriage at delivery.

**Conclusion:**

These findings suggest that both high coverage of IPTp-SP and the use of AL for the treatment of malaria infection/disease during pregnancy select for *pfmdr1* N86 wild-type allele at delivery.

## Background

Malaria in pregnancy (MiP) is a significant public health problem, with substantial adverse effects on both mother and fetus, including maternal anaemia, fetal loss, premature delivery, intrauterine growth retardation, and delivery of low birth-weight infants, which is a risk factor for death [1–4]. MiP current control measures used in most endemic countries, according to WHO recommendations, include the use of insecticide treated nets (ITNs), intermittent preventive treatment with sulfadoxine-pyrimethamine (IPTp-SP) and effective case management of malaria, which, since 2010, includes the use of artemisinine-based combination therapy (ACT) [5, 6].

Current efforts of malaria control during pregnancy rely mostly on the effectiveness of anti-malarial drugs used for both IPTp and case management. Indeed, although there are other limiting factors, including low attendance rate of antenatal services [7–10], low coverage and compliance to the preventive treatment by pregnant women [11–13] and inadequate protection of fewer than three SP doses where malaria transmission is intense [12, 14], the major hindrance of the effectiveness of IPTp-SP policy is the spread of *Plasmodium falciparum* resistance to SP [15–20]. Given the number of studies reporting the increased resistance of *P. falciparum* to SP, there has been several responses: (i) the increase of IPTp-SP doses by WHO in 2012 [21]; (ii) the evaluation of alternative drugs to SP [22, 23]; and, (iii) the assessment of alternative or improved strategies to IPTp-SP [24]. However, none of the alternative drugs or strategies tested has prompted the replacement of the IPTp-SP policy [25, 26]. Reducing the burden of malaria during pregnancy in high transmission setting remains challenging.

Since 2015, WHO recommends the use of ACT for the treatment of *P. falciparum* uncomplicated malaria during the second and third trimester of pregnancy [27, 28], and such recommendation has already been adopted and implemented by all sub-Saharan African countries [29]. However, there is limited knowledge on the effect of ACT, treatments such as artemether-lumefantrine (AL), on the selection of *P. falciparum* resistance markers during pregnancy that can affect the treatment outcome. Indeed, available information in non-pregnant women have shown that the *P. falciparum* multidrug resistance 1 (*pfmdr1)* N86 and D1246 alleles might be associated with AL resistance [30, 31]. In addition, the combination of N86, 184F and D1246, forming the ‘NFD’ haplotype, led to a decreased susceptibility to AL and treatment with AL selects for such a haplotype [32–34]. There is therefore a need for pharmacovigilance studies to monitor any delayed parasite clearance by AL and to assess risk factors associated with the carriage of *P. falciparum* resistance markers.

Between 2013 and 2016, a multi-centre, cluster-randomized, controlled trial (COSMIC) was conducted in three West African countries with high (Burkina Faso, Benin) and low (The Gambia) malaria transmission, to assess the protective efficacy of adding community-scheduled screening and treatment of malaria during pregnancy (CSST) to standard IPTp-SP (CSST/IPTp-SP) [35, 36]. The CSST/IPTp-SP strategy was based on monthly active follow-up by community health workers using rapid diagnostic tests (RDTs). The aim of the combined CSST/IPTp-SP strategy was to provide an opportunity to detect and treat malaria infections during pregnancy with AL and reduce the prevalence of placental malaria [35]. As part of the COSMIC trial, it has shown a high prevalence of the triple *dhfr* mutation with presence of quintuple mutants (triple *dhfr* and double *dhps*) in Burkina Faso, confirming concerns about the efficacy of IPTp-SP in the near future [37]. This study aimed to determine the prevalence and factors associated with the carriage of *pfmdr1* polymorphisms (*pfmdr1* N86Y7, Y184F, D1246Y) among pregnant women within the COSMIC trial in Burkina Faso.

## Methods

### Study site

This study was conducted in Nanoro Health District (NHD) in Burkina Faso between 2013 and 2016. NHD is a rural setting located in the centre-west region of the country, 85 km from the capital city, Ouagadougou. Malaria transmission in the region is endemic and highly seasonal. There is year-round malaria transmission with high transmission season occurring from July to December, corresponding to the wet period in the country. Most malaria cases are due to *P. falciparum*.

### Study participants and sample size

The samples analysed in this study were collected from pregnant women enrolled in a clinical trial assessing the effectiveness of MiP preventive treatments, known as COSMIC trial (Clinical Trials.gov Identifier: NCT1941264). In the COSMIC trial, the protective efficacy of adding CSST during pregnancy to the standard IPTp-SP (CSST/IPTp-SP, intervention arm) was compared to IPTp-SP alone (control arm) [35]. CSST intervention was implemented by community health workers through monthly screening using RDTs and treatment of malaria infection with AL. AL treatment was given for both malaria episodes and infections detected by RDTs in the intervention arm, whereas in the control arm (standard IPTp-SP alone), AL was given for clinical episode treatment only, according to the national guidelines.

**Fig. 1:**
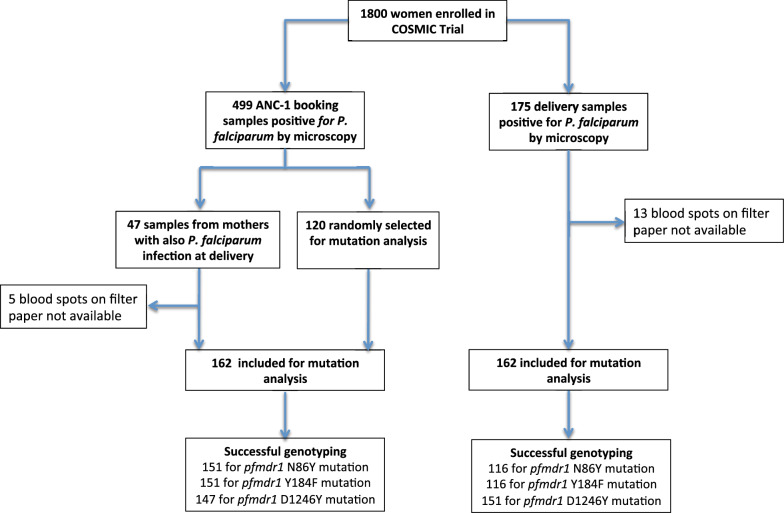
Flow diagram of samples selection

For the present analysis, a total of 324 dried blood spots (DBS) collected from finger prick at enrolment (first antenatal care visit (ANC-1)) and at delivery were selected from participants in Burkina Faso based on light microscopy (LM) results as shown in Fig. 1: (i) all available DBS collected at delivery from pregnant women with a *P. falciparum* infection (N = 162); (ii) all available DBS collected at ANC-1 from pregnant women who experienced *P. falciparum* infection both at recruitment and delivery (N = 42); and, (iii) a random selection of DBS collected at ANC-1 from pregnant women with a *P. falciparum* infection (N = 120).

## Laboratory methods

### DNA extraction

*Plasmodium falciparum* genomic DNA was extracted using the QIAamp 96 DNA Blood Kit (Qiagen, Germany) following the manufacturer’s instructions.

### PCR-RLFP

*pfmdr1* 86Y, 184F and 1246Y mutations were determined using nested PCR followed by restriction fragment length polymorphism (RFLP) analysis as previously described [38]. PCR products were digested with *Afl*III (NEB), *Dra*I (NEB), and *Eco*RV (NEB) for the determination of *pfmdr1* N86Y, Y184F, and D1246Y alleles, respectively (Table 1). The digested products were visualized by electrophoresis using 2.5% agarose gel for 2 h at 80 V. The nested PCR and digestion reactions were run using 3D7 as wild-type control for the three SPNs and V1 (for N86Y) and 7G8 (for Y184F and D1246Y) as mutant controls.

**Table 1:** Primer sequences used for the nested PCR and restriction enzymes used for the RLFP

Primer name	Sequence	Restriction enzyme
Nested1 forward Pfmdr1 86 (MDR-A1)	TGTTGAAAGATGGGTAAAGAGCAGAAAGAG	*Afl*III (NEB)
Nested1 reverse Pfmdr1 86 (MDR-A3)	TACTTTCTTATTACATATGACACCACAAACA	
Nested2 forward Pfmdr1 86 (MDR-A4)	AAAGATGGTAACCTCAGTATCAAAGAAGAG	
Nested2 reverse Pfmdr1 86 (MDR-A2)	GTCAAACGTGCATTTTTTATTAATGACCAttTA	
Nested1 forward Pfmdr1 184 (MDR-A1)	TGTTGAAAGATGGGTAAAGAGCAGAAAGAG	*Dra*I (NEB)
Nested1 reverse Pfmdr1 184 (MDR-A3)	TACTTTCTTATTACATATGACACCACAAACA	
Nested2 forward Pfmdr1 184 (MDR-A4)	AAAGATGGTAACCTCAGTATCAAAGAAGAG	
Nested2 reverse Pfmdr1 184 (MDR-A2)	GTCAAACGTGCATTTTTTATTAATGACCAttTA	
Nested1 forward Pfmdr1 1246 (MDR-O1)	AGAAGATTATTTCTGTAATTTGATACAAAAAGC	*Eco*RV (NEB)
Nested1 reverse Pfmdr1 1246 (MDR-O2)	ATGATTCGATAAATTCATCTATAGCAGCAA	
Nested2 forward Pfmdr1 1246 (1246F)	ATGATCACATTATATTAAAAAATGATATGACAAAT	
Nested1 reverse Pfmdr1 1246 (MDR-O2)	ATGATTCGATAAATTCATCTATAGCAGCAA	

### Sulfadoxine-pyrimethamine resistance markers

The assessment of *P. falciparum* mutations in the *dhfr* gene (codons N51, C59, S108) and the *dhps* gene (codons A437, K540) was performed as part of the COSMIC trial [35, 37] and, re-analysed in this manuscript. The *dhfr* and *dhps* genes were amplified by nested PCR and products were sequenced to identify the targeted mutations as previously reported [37].

## Statistical analysis

Data were analysed using STATA version 14.0 (StataCorp, USA). The *pfmdr1* genotype profile was determined according to the presence or absence of wild/mutant alleles. Samples in which both wild and mutant alleles were detected were considered as mutant allele carriers. Differences between samples collected at ANC-1 and at delivery were estimated using the Chi-square test for proportions. Factors associated with *pfmdr1* 86Y mutant allele carriage were assessed by univariate and multivariable logistic regression analyses. Variables with *P* values ≤ 0.10 in univariate analyses were included in the multivariable models. The investigated variables included: MiP preventive strategy, parasitaemia, age, gravidity, number of malaria episodes, number of IPTp-SP doses received during pregnancy, AL treatment during pregnancy, haemoglobin levels at delivery, and bed-net usage. *P* values less than 0.05 were considered statistically significant.

## Results

### Characteristics of study population

The characteristics of the study participants are shown in Table 2. The mean age of pregnant women infected both at ANC-1 and at delivery (21.5 ± 5.6 years) was significantly lower than that of women infected only at ANC-1 (23.9 ± 5.7 years) and of women infected only at delivery (26.5 ± 6.1 years) (*P* < 0.001). The median parasite density was 980.5 (IQR: 412.5–2,949.2) parasites/µl and 2,237.2 (IQR: 613.5–11,425.7) parasites/µl at ANC-1 and at delivery, respectively (*P* = 0.02). Among women infected at ANC-1, the median parasite density was significantly higher among those infected both at ANC-1 and at delivery (1,752.5 (IQR: 558.5–5,373.5) parasites/µl) compared to those infected only at ANC-1 (903 (IQR: 324.5–2,308.5) parasites/µl; *P* = 0.01). Among women infected at delivery, there was no significant difference of the median parasite density between those infected only at delivery and those infected both at ANC-1 and at delivery (*P* = 0.84). There was no significant difference of the proportion of women who benefited from CSST intervention between the three groups (*P* = 0.40). The proportion of primigravid and secondigravid women infected both at ANC-1 and at delivery was significantly higher than that of women infected only at ANC-1 and those infected only at delivery (*P* < 0.001).

**Table 2:** Characteristics of study population

Characteristics	Women infected at ANC-1 (enrolment) (N = 120)	Women infected at Delivery (N = 120)	Women infected at ANC-1 and at delivery (N = 42)	*P*
Age at enrolment (years, mean ± SD)	23.9 (±5.7)	26.5 (± 6.1)	21.5 (5.6)	< 0.001
Parasite density by LM (parasites/μl, Median (IQR))				
ANC-1	903 (324.5–2308.5)	–	1752.5 (558.5–5373.5)	0.01
Delivery	–	1955.7 (565.2–9733)	2268 (835–15,485)	0.84
MiP preventive strategy				
IPTp-SP	67 (55.8)	58 (48.3)	20 (47.6)	0.40
CSST/IPTp-SP	53 (44.2)	62 (51.7)	22 (52.4)	
Gravidity				
≤ 2	63 (52.5)	35 (29.2)	32 (76.2)	< 0.001
> 2	57 (47.5)	85 (70.8)	10 (23.8)	
ITN usage^a^				
Night before ANC-1	103 (92.8)	–	24 (72.7)	0.004
Night before delivery	–	69 (62.2)	24 (72.7)	0.30
IPTp-SP doses during pregnancy				
< 3	–	82 (68.3)	33 (78.6)	0.21
≥ 3	–	38 (31.7)	9 (21.4)	
AL treatment during pregnancy				
0	–	67 (55.8)	13 (31.0)	0.02
1	–	33 (27.5)	19 (45.2)	
≥ 2		20 (16.7)	10 (23.8)	
Timing AL treatment to delivery				
No treatment		67 (55.8)	13 (31.0)	0.05
4 to 28 days	–	18 (15.0)	8 (19.0)	
29 to 42 days	–	13 (10.8)	10 (23.8)	
43 to 63 days		13 (10.8)	5 (11.9)	
≥ 64 days		9 (7.5)	4 (14.3)	
Haemoglobin level at delivery^a^				
< 11 g/dl	–	63 (53.4)	20 (50.0)	0.72
≥ 11 g/dl	–	55 (46.6)	20 (50.0)	
Malaria episode during pregnancy				
≤ 2	–	58 (48.3)	0 (0.0)	< 0.001
> 2	–	62 (51.7)	42 (100)	
Triple *dhfr* 51/59/108 mutation at delivery^a^				
No		19 (28.8)	6 (35.3)	0.60
Yes		46 (71.2)	11 (64.7)	

^a^ Missing: ITN usage (women infected only at ANC-1 (N = 111), women infected only at delivery (N = 111), Women infected both at ANC-1 and at delivery (N = 33)); Haemoglobin level at delivery (women infected only at delivery (N = 118), Women infected both at ANC-1 and at delivery (N = 40)); Triple *dhfr* 51/59/108 mutation (Women infected only at delivery (N = 66), Women infected both at ANC-1 and at delivery (N = 17)

Among pregnant women infected at ANC-1, there was a significant difference of bed-net use between those infected both at ANC-1 and at delivery (72.7% (24/42)) and those only infected at ANC-1 (92.8% (103/120); *P* = 0.004). Such a difference was not observed among women infected at delivery (*P* = 0.30). Among pregnant women who experienced malaria infection at delivery, 69% (29/42) of those infected both at ANC-1 and at delivery received at least one AL treatment during pregnancy against 44.2% (53/120) of those infected only at delivery (*P* = 0.02). There was a significant difference of the time of AL treatment to delivery between women infected only at delivery and that of women infected both at ANC-1 and at delivery (*P* = 0.05). The proportion of women who received at least three IPTp-SP doses was not significantly different between those infected only at delivery and that of those infected both at ANC-1 and at delivery (*P* = 0.21). The carriage of triple *dhfr* 51/59/108 mutation was similar among women infected only at delivery and those infected both at ANC-1 and at delivery (*P* = *0.60*). Only two women were found to carry a double *dhps* 437/540 mutant allele at delivery. Of note, data on *dhfr*-*dhps* mutations among the study popualtion were obtained from the main COSMIC trial [36, 37] and re-analysed in this manuscript.

### Plasmodium falciparum resistance markers genotyping success rate

In total, the success rate for *pfmdr1* D1246Y genotyping was 92.0% (298/324), with rates of 90.7% (147/162) and 93.2% (151/162) for samples collected at ANC-1 and at delivery, respectively. The *pfmdr1* Y184F and N86Y genotyping showed similar success rates as the samples underwent the same nested PCR procedure. The success rate for the samples collected at ANC-1 was 93.2% (151/162), while the success rate for the samples collected at delivery was 71.6% (116/162), giving a total success rate of 82.4% (267/324) for both Y184F and N86Y codons.

### Prevalence of *pfmdr1* alleles among the study population

The mutant *pfmdr1* 86Y allele was detected among the study participants with a prevalence of 13.2% (20/131) and 12.1% (14/116) at ANC-1 and at delivery, respectively (*P* = 0.77). Among women who experienced malaria infection both at ANC-1 and at delivery, the prevalence of the mutant *pfmdr1* 86Y allele was 7.9% (3/38) and 16.1% (5/31) at ANC-1 and at delivery, respectively (*P* = 0.25). There was no significant difference of the proportion of *pfmdr1* 86Y allele carriage between women infected at ANC-1 and those infected at delivery for both women who received the standard IPTp-SP treatment (*P* = 0.63) and those who received the CSST/IPTp-SP treatment (*P* = 0.38). By contrast, no mutant alleles corresponding to *pfmdr1* Y184F and D1246Y codons were observed in the samples collected from either ANC-1 or delivery (Table 3).

**Table 3:** Prevalence of *pfmdr1* N86Y alleles among the study population

Codon	N86 allele		86Y allele		N86 + 86Y alleles		Total 86Y allele carriage		P*
	n	% (95%CI)	n	% (95%CI)	n	% (95%CI)	n	% (95%CI)	
Total study population									
ANC-1 (N = 151)	131	86.8 (80.3–91.7)	5	3.3 (1.1–7.6)	15	9.9 (5.7–15.9)	20	13.2 (8.3–19.7)	0.77
Delivery (N = 116)	102	87.9 (80.6–93.2)	2	1.7 (0.2–6.1)	12	10.3 (5.5–17.4)	14	12.1 (6.8–19.4)	
Women infected at ANC-1 and at delivery									
ANC-1 (N = 38)	35	92.1 (78.6–98.3)	1	2.6 (0.1–13.8)	2	5.3 (0.6–17.8)	3	7.9 (1.7–21.4)	0.25
Delivery (N = 31)	26	83.9 (66.3–94.5)	0	0	5	16.1 (5.4–33.7)	5	16.1 (5.4–33.7)	
Women who received standard IPTp-SP									
ANC-1 (N = 81)	73	90.1 (81.5–95.6)	1	1.2 (0.03–6.7)	7	8.7 (3.6–17.0)	8	9.9 (4.4–18.5)	0.63
Delivery (N = 56)	49	87.5 (75.9–94.8)	2	3.6 (0.4–12.3)	5	8.9 (3.0–19.6)	7	12.5 (5.2–24.1)	
Women who received CSST/IPTp-SP									
ANC-1 (N = 70)	58	82.9 (72.0–90.8)	4	5.7 (1.6–14.0)	8	11.4 (5.1–21.3)	12	17.1 (9.2–28.0)	0.38
Delivery (N = 60)	53	88.3 (77.4–95.2)	0	0	7	11.7 (4.8–22.6)	7	11.7 (4.8–22.6)	

^*^P value for total mutant (86Y) allele carriage *versus* wild-type (N86) allele carriage

### Factors associated with *pfmdr1* 86Y mutant allele carriage at ANC-1 and at delivery

Univariate and multivariable logistic regression analyses were performed to assess the factors associated with the *pfmdr1* 86Y mutant allele at ANC-1 and at delivery (Tables 4 and 5). None of the variables investigated in the univariate analysis was significantly associated with the carriage of *pfmdr1* 86Y mutant allele at ANC-1 (*P* > 0.1). Consequently, no multivariable analysis was undertaken for further assessment (Table 4). At delivery, univariate analyses showed a tendency of an increase risk of *pfmdr1* 86Y mutant allele carriage with parasite density above the median (*P* = 0.08). By contrast, there was a tendency towards reduced risk of *pfmdr1* 86Y mutant allele carriage among women who received at least three IPTp-SP doses during pregnancy (*P* = 0.06) and those who received at least one AL treatment during pregnancy (*P* = 0.10). In multivariable analyses, these associations were confirmed as infections at delivery with a parasite density more than the median (2,237.2 (IQR: 613.5–11,425.7) parasites/µl) was associated with an increase risk of *pfmdr1* 86Y mutant allele carriage (AOR = 5.5 (95% CI 1.07–28.0); *P* = 0.04). In addition, both three or more IPTp-SP doses (AOR = 0.25 (95% CI 0.07–0.92); *P* = 0.04) and one or more AL treatment (AOR = 0.25 (95% CI 0.07–0.89); *P* = 0.03) during pregnancy were associated with a significant reduce risk of *pfmdr1* 86Y mutant allele carriage at delivery. None of the other variables of interest, including MiP preventive strategy, triple *dhrf* 51/59/108 mutation, timing of AL treatment to delivery, gravidity, and malaria infection both at ANC-1 and at delivery, showed a significant association with *pfmdr1* 86Y mutant allele carriage (Table 5).

**Table 4:** Univariate analyses assessing factors associated with *pfmdr1* 86Y mutant allele at ANC-1

Variables	N	OR (95%CI)	*P*
Parasite density	151		
< Median (980.5 parasites/µl)	71	1	
≥ Median	80	1.39 (0.53–3.62)	0.50
MiP preventive strategy	151		
IPTp-SP	81	1	
CSST/IPTp-SP	70	1.89 (0.72–4.92)	0.19
Age	151		
< Mean (23 years ± 6.1)	66	1	
≥ Mean	85	1.28 (0.50–3.29)	0.61
Gravidity	151		
Primi-secundigravidae	92	1	
Multigravidae	59	1.32 (0.51–3.42)	0.56
ITN usage	136		
No	14	1	
Yes	122	0.97 (0.20–4.73)	0.97

**Table 5:** Univariate and multivariable analyses assessing factors associated with *pfmdr1* N86Y mutant allele at delivery

Variables	N	OR (95%CI)	*P*	AOR (95%CI)	*P*
Parasite density	116				
< Median (2,237.2 parasites/µl)	43	1			
≥ Median	73	4.03 (0.86–19.0)	0.08	5.5 (1.07–28.0)	0.04
Number of SP doses	116				
< 3	78	1			
≥ 3	38	0.13 (0.02–1.07)	0.06	0.25 (0.07–0.92)	0.04
MiP preventive strategy	116				
IPTp-SP	56	1			
CSST/IPTp-SP	60	0.92 (0.30–2.83)	0.89		
Triple *dhfr* 51/59/108 mutation					
No	25	1			
Yes	58	1.64 (0.30–8.95)	0.57		
AL treatment during pregnancy					
0	51	1			
≥ 1	65	0.39 (0.12–1.24)	0.10	0.25 (0.07–0.89)	0.03
Timing Al treatment to delivery					
No treatment	51	1			
4 to 28 days	20	0.51 (0.10–2.64)	0.43		
29 to 42 days	21	0.001 (0.001–1.01)	0.96		
43 to 63 days	13	0.39 (0.04–3.38)	0.39		
≥ 64 days	11	1.04 (0.19–5.64)	0.97		
Age	116				
< Mean (25.2 years ± 6.4	58	1			
≥ Median	58	0.88 (0.28–2.71)	0.82		
Gravidity	116				
Primi-secundigravidae	55	1			
Multigravidae	61	0.64 (0.21–1.98)	0.44		
Malaria episode	116				
< 2	37	1			
≥ 2	79	0.82 (0.25–2.65)	0.74		
Malaria infection at ANC-1 and at delivery					
No	85	1			
Yes	31	1.62 (0.50–5.28)	0.42		
Haemoglobin level at delivery	113				
≥ Mean 11.5 g/dl ± 1.7	55	1			
< Mean	58	0.68 (0.22–2.10)	0.50		
ITN usage	101				
No	34	1			
Yes	67	0.46 (0.13–1.55)	0.21		

## Discussion

Despite the widespread implementation of IPTp-SP to prevent MiP, pregnant women in endemic countries often experience peripheral and/or placental malaria infection at delivery [15, 39, 40]. Although WHO revised IPTp-SP guidelines and increased the SP dose, which has been shown to improve birth outcomes [41, 42], IPTp-SP strategy is still threatened by increasing *Plasmodium falciparum* resistance to SP. Consequently, there is a need to develop new alternative or improved strategies as part of IPTp-SP policy. In line with the latter, CSST of MiP, in addition to standard IPTp-SP (CSST/IPTp-SP), was tested in Burkina Faso, Benin and The Gambia as an intervention to improve maternal health and birth outcomes in areas of different malaria transmission intensity (COSMIC trial, NCT1941264) [35]. In such a context, the current study was conducted to determine prevalence and factors associated with the carriage of *pfmdr1* polymorphisms among pregnant women participating in the COSMIC trial in Burkina Faso.

Mutations in the gene-encoding *pfmdr1* are known to be associated with aminoquinoline resistance [43], and therefore represent key *P. falciparum* markers for monitoring resistance in both susceptible groups (children under 5 years and pregnant women) and the general population. In this study, the analysis was focused on: (i) mutations in *pfmdr1* N86Y and D1246Y codons, which have been associated with resistance to chloroquine and amodiaquine [44–46], whereas wild-type sequences in these alleles were associated with reduced sensitivity to lumefantrine [44, 47, 48]; and, (ii) mutation in the *pfmdr1* Y184F codon, which was associated with altered sensitivity to artemisinins and mefloquine [49]. A prevalence of 13.2 and 12.1% of the *pfmdr1* 86Y mutant allele was found at ANC-1 and at delivery, respectively. No mutant allele was observed for *pfmdr1* Y184F and *pfmdr1* D1246Y codons at both ANC-1 and at delivery. The observed prevalence of these mutations at positions 86, 184 and 1246 in this study lack comparable data in the country as previous studies differ with regard to study population (children *vs* adults *vs* pregnant women), *Plasmodium falciparum* isolates (clinical *vs* asymptomatic infections), and study periods [50–53]. However, looking at available reports in the study area, these results showed a higher prevalence of the *pfmdr1* 86Y mutant allele in pregnant women at ANC-1 (13.2%) compared to that in patients with uncomplicated malaria two years before (pre-treatment prevalence of 8.3% in 2010–2012) [50]. An early study conducted by the time of adoption of ACT as a first-line treatment for uncomplicated falciparum malaria in the country (2005), reported a higher prevalence of *pfmdr1* 86Y mutant allele (35–40%) in children aged 6–59 months with uncomplicated malaria [51]. Surprisingly, no *pfmdr1* 184F mutant allele was detected among the study population while a prevalence of about 50% was reported among *P. falciparum* uncomplicated malaria patients in 2010–2012 [50]. In the same study, only three *pfmdr1* 1246Y mutant alleles were detected in 660 isolates, corresponding to a prevalence of 0.4%.

It has been shown that wild-type sequences of *pfmdr1* N86Y, Y184F and D1246Y codons are selected by prior use of AL treatment in malaria patients [47, 48, 51–53]. By contrast, selection of the *pfmdr1* 184F mutant allele has been observed in prior therapy with AL in malaria patients in Uganda [47]. To explore the potential selection of wild-type/mutant sequences of *pfmdr1* polymorphisms following AL treatments during pregnancy, the prevalence of *pfmdr1* 86Y mutant alleles at ANC-1 was compared to that at delivery. No significant difference of the *pfmdr1* 86Y mutant allele was found neither in the general study population (*P* = 0.77) nor in sub-groups represented by women infected both at ANC-1 and at delivery (P = 0.25), women who received the standard IPTp-SP (*P* = 0.63) and women who benefitted from additional screening and treatment of *P. falciparum* infections (asymptomatic infections) using AL (*P* = 0.38).

To further assess the potential selection of *pfmdr1* N86Y mutant/wild-type alleles by specific factors, factors associated with *pfmdr1* 86Y mutant allele carriage at ANC-1 and at delivery were investigated. Among the variables evaluated, none was significantly associated with *pfmdr1* 86Y mutant allele carriage at ANC-1. However, *Plasmodium falciparum* infection at delivery with high parasitaemia was significantly associated with nearly 5.5 times increase risk of *pfmdr1* 86Y mutant allele carriage at delivery after adjusting by confounding factors (*P* = 0.04). By contrast, uptake of at least three IPTp-SP doses and at least one AL treatment were found to be significant protective factors against *pfmdr1* 86Y mutant allele carriage at delivery in multivariate analyses (75% reduction for both with *P* = 0.04 and *P* = 0.03, respectively). These results suggest a benefit in reducing the risk of aminoquinoline resistance marker carriage by high coverage of IPTp-SP doses, which has been shown to reduce parasite load in *P. falciparum* infection during pregnancy [54, 55]. Moreover, these findings suggest a positive selection of *pfmdr1* N86 wild-type allele at delivery following AL treatment during pregnancy in women receiving IPTp-SP, similar to that reported in non-pregnant women in Southeast Asia (Thailand) and East Africa (Kenya) [30, 31] and in patients of all age groups in West Africa (Burkina Faso) [50].

Studies have demonstrated that recent AL use has more of an impact on *pfmdr1* N86 wild-type allele prevalence than less recent AL use, as lumefantrine levels decline over time, resulting in less selection [56, 57]. On the other hand, it has been shown that AL could select for *pfmdr1* N86 wild-type allele a few months post-treatment in children [58]. This could be explained by genetic variations or other factors including the administration of AL with fatty foods, leading some individuals to exhibit longer artemether or lumefantrine half-lives than other, allowing longer periods of selection [58, 59]. Although, no evidence of an impact of the timing of AL treatment to delivery on the selection of *pfmdr1* N86 wild-type allele was observed in this study, the existence of a specific selective window in pregnant women should not be ruled out given the limited number of *pfmdr1* 86Y mutant allele carriers. In addition, the sub-group of women who experienced malaria infection both at ANC-1 and at delivery did not show a significant association with *pfmdr1* 86Y mutant allele carriage at delivery and no significant difference was found for triple *dhfr* 51/59/108 mutations carriage in this sub-group compared to those infected only at delivery (*P* = *0.6*), suggesting new infection parasite population after IPTp-SP and eventually AL treatments. In this regard, the lack of *Plasmodium falciparum* genotyping to distinguish recrudescent parasites to new infection parasites could be seen as another limit of this study. Future investigations on factors associated with *pfmdr1* gene polymorphisms selection in pregnant women living in endemic countries should include large sample size and parasites population genotyping.

## Conclusion

This study showed that the uptake of three or more IPTp-SP doses and one or more AL treatments are significantly associated with a reduced risk of *pfmdr1* 86Y mutant allele carriage in pregnant women at delivery. These findings suggest that both high coverage of IPTp-SP and the use of AL for the treatment of malaria infection/disease during pregnancy select for *pfmdr1* N86 wild-type allele at delivery.

## Data Availability

All data generated or analyzed during this study are included in this published article.

## Abbreviations

ANC-1: First antenatal care
MiP: Malaria in pregnancy
IPTp-SP: Intermittent preventive treatment during pregnancy with sulfadoxine-pyrimethamine
CSST: Community-based scheduled screening and treatment of malaria during pregnancy
ITN: Insecticide treated net
IQR: Interquartile range
LM: Light microscopy
OR: Odds ratio
AOR: Adjusted odds ratio
